# Possibility to Estimate Same Day Energy Status of Dairy Cows during First Half of Lactation by Non-Invasive Markers with Emphasis to Milk Fatty Acids

**DOI:** 10.3390/ani13142370

**Published:** 2023-07-20

**Authors:** Tiia Ariko, Tanel Kaart, Katri Ling, Merike Henno, Hanno Jaakson, Meelis Ots

**Affiliations:** 1 Chair of Animal Nutrition, Institute of Veterinary Medicine and Animal Sciences, Estonian University of Life Sciences, Fr. R. Kreutzwaldi 46, 51006 Tartu, Estonia; tiia.ariko@emu.ee (T.A.); merike.henno@emu.ee (M.H.); hanno.jaakson@emu.ee (H.J.); meelis.ots@emu.ee (M.O.); 2 Chair of Animal Breeding and Biotechnology, Institute of Veterinary Medicine and Animal Sciences, Estonian University of Life Sciences, Fr. R. Kreutzwaldi 1, 51006 Tartu, Estonia; tanel.kaart@emu.ee

**Keywords:** energy balance prediction, body condition score, biomarker, milk composition, fatty acids ratio

## Abstract

**Simple Summary:**

Negative energy balance experienced by dairy cows postpartum can cause problems to the animal and financial loss to the farmer. Therefore, a wide array of invasive and non-invasive methods for assessing the energy status of dairy cows have been developed. The present study compares the predictive ability of milk fatty acids and traditional indirect markers and suggests that milk fatty acid composition may be the potential sole predictor of the energy status in dairy cows.

**Abstract:**

Postpartum negative energy balance (NEB) is detrimental to cows and decreases profitability in dairy farming. The two origins of milk fatty acids (FA), de novo synthesized in the mammary gland and plasma lipids initially originating from feed, rumen microbes and the animal’s adipose tissue, make milk FA candidates as possible NEB biomarkers. The aim of this study was to assess the possibility to predict EB in cows in the first 150 days of lactation with BCS, milk traits and selected individual milk FA and the ratios of blood-derived and de novo synthesized FA. The daily EB of Estonian Holstein cows (N = 30) was calculated based on body weights and BCS values. Milk FA were analyzed with gas chromatography. The variance partitioning analysis revealed that milk production traits, BCS at calving, FA ratios and days in milk accounted for 67.1% of the EB variance. Random forest analysis indicated the highest impact of the ratios C18:1cis9/C12:0+C14:0, C18:1cis9+C18:0/C12:0+C14:0, C18:1cis9/C14:0, C18:1cis9+C18:0/C14:0, C18:1cis9/sum C5:0 to C14:0, C18:1cis9+C18:0/sum C5:0 to C14:0 or C18:1cis9/C15:0. FA and their ratios alone explained 63.6% of the EB variance, indicating the possibility to use milk FA and their ratios as sole predictors for the energy status in dairy cows.

## 1. Introduction

Postpartum negative energy balance (NEB) in dairy cows is characterized by elevated blood nonesterified fatty acids (NEFA) due to adipose tissue mobilization. Excessive synthesis of ketone bodies in the liver in the course of incomplete oxidation of NEFA could lead to ketosis and a wide array of problems for the animal (different health issues, reduced fertility, etc.) but also decrease the profitability of the dairy farm; hence, the issue has been widely researched (reviewed by [1]). As the length and depth of NEB are important indicators that problems depend on, EB data would be helpful for identifying potential problems and making management decisions [2]. As the direct assessment of cows’ EB on a commercial farm is not feasible, a lot of attention has been given to finding indirect measures of energy balance (EB) in individual animals. Body condition scoring [3], milk fat to protein ratio [4,5], blood NEFA and ketone bodies [6,7] have been identified and used as biomarkers of NEB.

Milk fatty acids (FA) originate from two sources: de novo synthesis in the mammary gland and plasma lipids initially originating from feed, rumen microbes and the animal’s adipose tissue [8]. The main FA in adipocytes are C18:1cis9 [9], C16:0 and C18:0 [10], which are released by lipolysis during NEB. The two origins, one related to body fat reserves reflecting the metabolic status of the animal, make milk FA potential candidates as NEB biomarkers.

Changes in the balance of C18:0, C18:1cis9 and de novo synthesized milk FA have been discussed as biomarker candidates for NEB in research papers [11,12]. Jorjong et al. [13,14] proposed that milk fat C18:1cis9 concentration measured in the second week of lactation could be used as an early warning for elevated blood plasma NEFA concentration, and the C18:1cis9 to C15:0 ratio may be a biomarker for hyperketonemia, both related to NEB. No previous long-term studies targeted the prediction of concurrent EB of dairy cows with different body condition scores (BCS) by different non-invasive markers, focusing on FA ratios based on their origin.

The hypothesis of this study was that the daily EB of dairy cows can be predicted by non-invasive markers, including milk FA concentrations and their ratios at the beginning of lactation during NEB and mid-lactation during positive EB. The aim was to assess the prediction strength of milk FA compared to other non-invasive indicators to estimate the daily EB of dairy cows and to find the individual FA and the ratios of blood-derived to de novo synthesized FA in the milk fat that can predict EB for every day in milk (DIM) in dairy cows.

## 2. Materials and Methods

### 2.1. Experimental Design

This experiment was conducted on the Eerika Experimental Farm of the Estonian University of Life Sciences (Märja, Estonia) with a herd size of 120 dairy cows, indoor freestall housing, TMR feeding and annual average milk production of 9200 kg.

This study was conducted on 30 Estonian Holstein cows (parity 2–6, BCS at calving 2.25–4.00) from one to three weeks before expected calving until approximately the 150th day of lactation.

The cows were fed grass silage, hay, maize meal, barley meal, heat-treated rapeseed cake and mineral feeds as TMR two times per day ad libitum at approximately 05:30 and 14:30 h. Depending on the physiological stage and requirements, the cows were fed 5 rations differing in chemical composition and nutritive value. The experimental diets were calculated according to the Estonian feeding recommendations: metabolizable energy (ME) according to Oll [15] and metabolizable protein (MP) based on the equations used in Finland [16] and fitted to suit Estonian conditions according to Kärt et al. [17]. Before calving and up to day 14 pre-partum, the far-off dry cow diet containing 8.7 MJ ME and 73 g MP/kg of DM was fed. From day 14 pre-partum until calving, the close-up dry cow diet containing 10.1 MJ ME and 87 g MP/kg of DM was offered. After calving until 6 days in milk (DIM), lactation diet one was provided, which was similar to the close-up diet differing only in its mineral composition. From 7 to 14 DIM, lactation diet two with an ME content of 10.9 MJ and MP content of 98 g per kg of DM was offered. Lactation ration three was fed from 15 DIM onwards containing 11.3 MJ ME and 104 g MP per kg of DM.

To calculate ration compositions, silage samples were taken twice weekly. Other feeds were sampled on a batch basis. All samples were analyzed for dry matter (DM), while chemical composition was analyzed using methods approved by the AOAC [18], and nutritive value (ME, MP) was calculated once weekly for the silage samples or on a batch basis for the other feeds. If necessary, during the experiment, the proportions of the rations’ ingredients were corrected to meet the required composition and nutritive values.

The body condition score (BCS) was registered [19] by two trained persons starting from four weeks before expected calving and during the whole period of the study with two-week intervals. The average number of BCS observations per cow was 7.3. The automatic daily weighting of the cows started on the 5.6th day after calving (min = 2, max = 22) on average and ended on the 152.6th day after calving (min = 104, max = 169) on average. Due to unforeseen problems, all daily weights were not available—the average number of daily weights per cow was 120.0 (min = 44, max = 164).

### 2.2. Sample Collection

The cows were milked in a DeLaval milking parlor twice a day at 05:00 and 15:00. Milk yields were recorded at each milking with DeLaval’s Alpro software version 6.5 for Windows (DeLaval International AB, Tumba, Sweden). Milk samples were collected by in-line milk meters (MM27BC, DeLaval International AB, Tumba, Sweden) twice weekly on Sundays and on Thursdays until confirmed pregnancy followed by once-a-week sampling on Sundays. Depending on calving and sampling day, the first sample was taken from 4 to 7 DIM. The last sampling day was aimed to be 150 DIM, but it was actually between 144 and 155 DIM. The average number of samples per cow was 41 (min 34, max 44). In total, 1231 milk samples were collected and analyzed. The samples from two consecutive milkings were pooled. A milk subsample (40 mL) of each pooled milk sample was preserved with bronopol (Broad Spectrum Microtabs, D&F Control Systems Inc., Dublin, CA, USA) and analyzed for fat, protein, lactose content and somatic cell count with an automatic infrared milk analyzer (System FT+, Foss Electric, Hillerod, Denmark) in the Milk Analysis Laboratory of Estonian Livestock Performance Recording Ltd. (Tartu, Estonia) The second subsample (1.5 mL) was stored at −20 °C until milk FA analyses. Milk fat was extracted according to Ariko et al. [20]. The fatty acid methyl esters (FAME) were analyzed using a gas chromatograph (Agilent 6890A, Agilent Technologies Inc., Santa Clara, CA, USA) equipped with a flame ionization detector and capillary column CP-Sil 88 (100 m × 0.25 mm i.d.) with a film thickness of 0.20 μm. Component FAME Mix (Supelco, Bellefonte, PA, USA), GLC 603 and GLC 408 (Nu-Chek Prep. Inc., Elysian, MN, USA), BAME Mix (Supelco, Bellefonte, PA, USA), Linoleic Acid Methyl Ester Isomer Mix (Supelco, Bellefonte, PA, USA) and individual fatty acid methyl ester standards were used to identify common FA. To identify the overlapping of trans and cis isomers, Ag+-SPE cartridges (750 mg/6 mL) (Supelco, Bellefonte, PA, USA) were used.

A total of 81 milk FA were identified; the studied FA and their ratios were selected according to their potential relationship with EB based on their origin and relation to body fat mobilization (Table 1).

### 2.3. Calculation of the Energy Balance of the Dairy Cows

**Figure A1:**
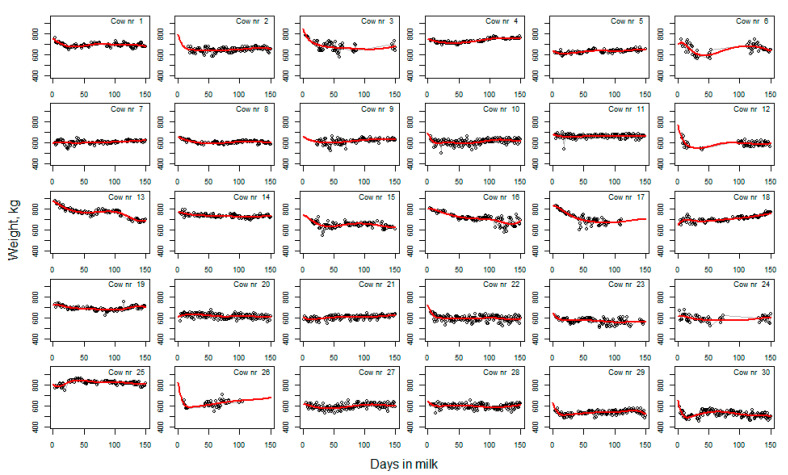
Actual weights (dots joined with grey line) and predicted daily weights (red lines) of the cows.

To predict the daily body weight (BW) of the cows, the general linear mixed model considered the cubic spline function of DIM with nonequidistant knots at 21, 70 and 112 DIM, parity and BCS group and their interactions with the cubic spline of DIM and the random cubic spline of DIM for each cow. The nonequidistant knots at DIM were selected by comparing the fits of different models: the Akaike information criterion (AIC) value of the final model was lower compared to the models with equidistant knots; models with knots at 12, 20, 60, 115 and 150 DIM (used by [21]); and models with less than three knots. The AIC of the model with four nonequidistant knots at 14, 21, 70 and 112 DIM was the lowest, but it was dropped as a visual inspection of the fitted curves showed that unrealistic weights in the first weeks for some cows with a lot of missing values were predicted. The parity by cubic spline and BCS group by the cubic spline interactions was included to fit different curves to potentially different groups of cows. The random cubic spline of DIM for each cow was considered to achieve cow-specific curves but avoid simultaneous perturbation by extreme outliers. The root mean square error of the fitted model was 19.1 kg (the modelling results are presented in Appendix A Figure A1).

**Figure A2:**
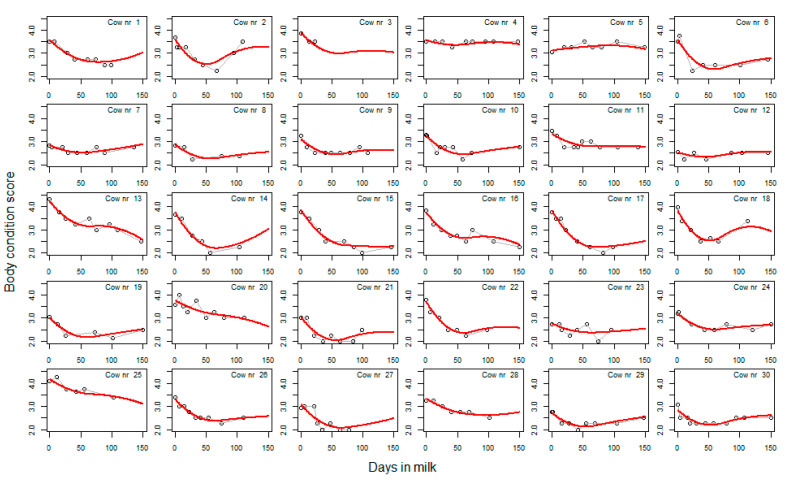
Body condition score values (dots joined with grey line) and predicted daily body condition score values (red lines) of the cows.

For the daily BCS prediction, the last pre-calving BCS was considered as the BCS at day 0. To predict the daily BCS, a similar model as for BW was used; however, the spline of degree 2 with one knot at 70 DIM was used. The root mean square error of the fitted model was 0.15 BCS units (the modelling results are presented in Appendix A Figure A2).

Both BW and BCS modelling were performed with an HPMIXED procedure in SAS 9.4 (SAS Institute Inc., Cary, NC, USA).

The daily EB of the cows in effective energy was estimated based on frequent BW measurements and BCS according to the approach of Thorup et al. [21]. In the following statistical analyses, only the EB values corresponding to DIM milk samples were used (the first days of lactation were omitted).

### 2.4. Statistical Analyses

Statistical analyses were performed and figures were constructed with statistical package R 3.3.3. The overall importance of FA and their ratios, milk production traits (milk yield, fat and protein percentage, lactose and fat-to-protein ratio, BCS at calving and DIM to predict the EB were studied, applying variance partitioning analysis (VPA; [22]) with function varpart in package vegan. To visualize the results of the VPA, the proportional Euler diagram was fitted using the function eulerr from the package eulerr.

Second, random forest was used to study more precisely the overall prediction ability of FA and their ratios and to find those with the strongest connection with the EB. Random forest was used because it allows for the consideration of possible nonlinear and interaction effects of different FA and their ratios. For prediction accuracy estimation, the leave-one-cow-out cross-validation approach was used. This means that the random forest was trained on a dataset without the data from one cow and then tested on the data of this cow to calculate the prediction errors and root mean square error (RMSE), and this approach was repeated for all cows. The importance of single FA and their ratios in random forest was calculated as the decrease in the residual sum of squares from splitting on the variable averaged over all trees. The random forests were applied with the function randomForest of the package with the same name.

Finally, to study the relationships of FA and their ratios with EB one by one, general linear mixed models were fitted. The models considered the natural cubic spline effect with one knot of FA or FA ratio and the random effect of the cow. In hypothesis testing, the Kenward–Roger approximation for the denominator degrees of freedom was used. The model accuracy was expressed as RMSE using the leave-one-cow-out cross-validation approach as described earlier. The packages lme4, lmerTest and splines were used, and the models were fitted with the function lmer.

As most of the FA and their ratios had right-skewed distributions, they were logarithm-transformed before analyses. This did not affect the results of the random forest analysis and had only a marginal effect on the VPA but made the general linear models in the cross-validation approach more stable.

## 3. Results

**Figure A3:**
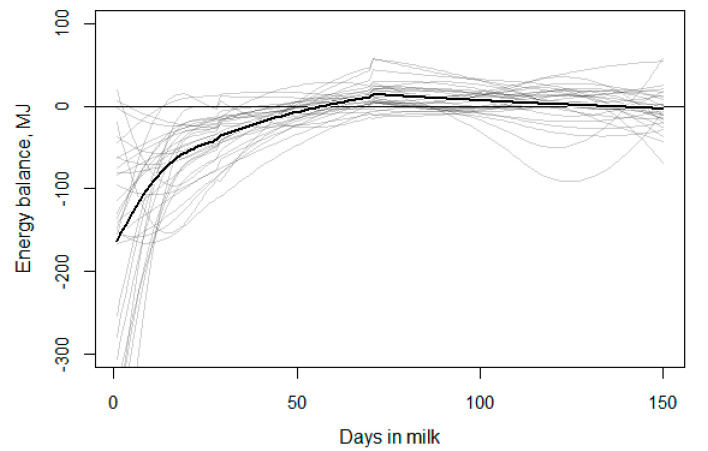
Predicted daily energy balances of the cows in effective energy (grey lines) and overall mean (bold black line).

The trends in the estimated EB curves of the cows followed the typical pattern of increasing in the first weeks of lactation in most of the cows with some exceptions. The depth and time of the nadir of NEB varied between the cows (Appendix A Figure A3).

**Figure 1:**
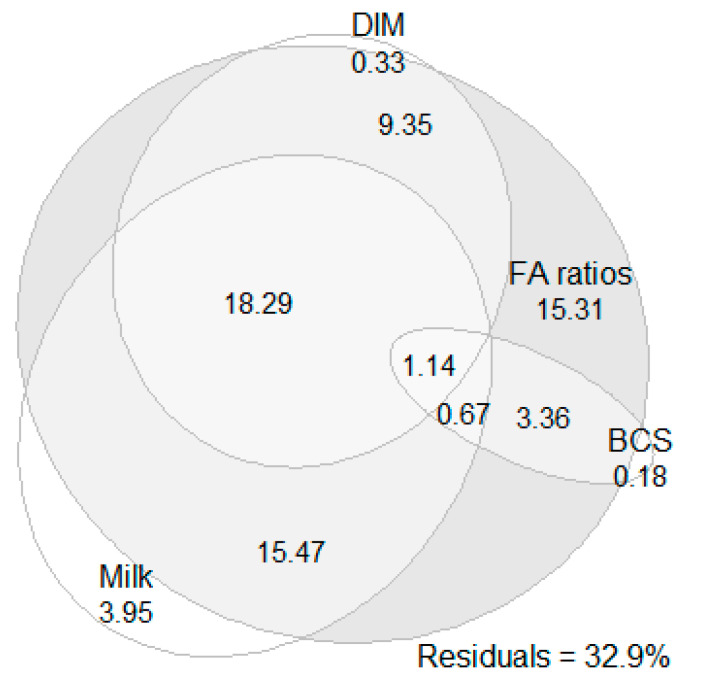
Results of variance partitioning analysis fitted with Euler diagram. The numerical values present the percentages of energy balance variance accounted for by milk production traits (milk yield, fat and protein percentage, lactose and fat-to-protein ratio), body condition score (BCS) at calving, milk fatty acids (FA) and their ratios (FA ratios; 26 ratios in total—see Table 1) and days in milk (DIM) and their intersections.

The results of the variance partitioning analysis showed that milk production traits, BCS at calving, FA and their ratios and DIM accounted for 67.1% of the EB variance. Moreover, FA and their ratios alone explained 63.6% of the EB variance, milk production traits alone explained 39.5% of the EB variance, DIM alone explained 29.0% of the EB variance, and BCS at calving alone explained 5.4% of the EB variance. The FA and their ratios’ effect covered almost totally the effects of DIM, milk production traits and BCS at calving and added an extra 15.3% of EB variance (Figure 1). The inclusion of the effect of the cow as a dummy variable increased the proportion of the EB variance that was accounted for by the factors above by only 3.7%.

**Figure 2:**
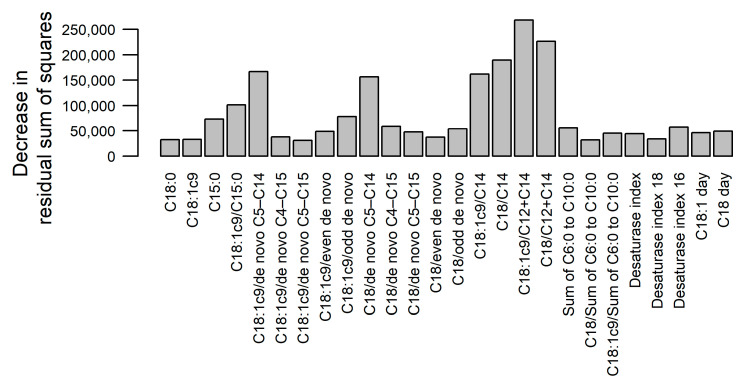
Results of random forest analysis: variables’ importance according to the mean decrease in residual sum of squares from splitting on the variable (higher bar indicates higher importance).

**Figure 3:**
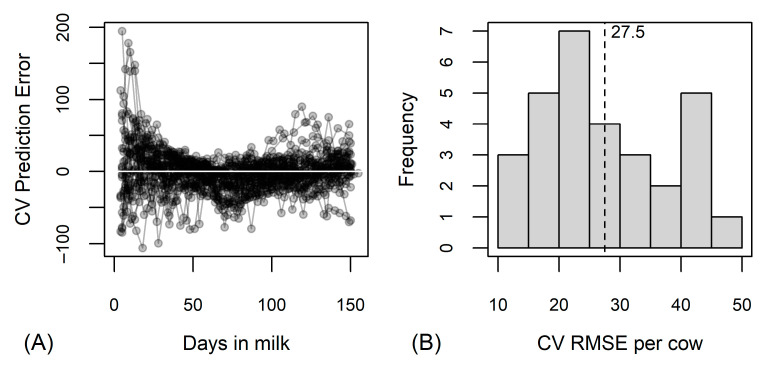
Results of random forest analysis predicting energy balance in MJ of effective energy based on 26 fatty acid traits. (**A**) Cross-validated (CV) prediction error by cows (one line corresponds to observations of one cow) and days in milk; (**B**) distribution of cross-validated root mean square errors (CV RMSE) per cow, vertical dotted line with numerical value denotes the average CV RMSE.

The random forest analysis indicated that the greatest decrease in prediction errors in the regression trees was achieved when splitting on the ratios C18:1c9/C12+C14, C18/C12+C14, C18:1c9/C14, C18/C14, C18:1c9/de novo C5–C14, C18/de novo C5–C14 or C18:1c9/C15 (Figure 2). The prediction errors were the greatest in the first weeks of lactation and in the last third of the study period (Figure 3A). The average cross-validated RMSE per cow was 27.6 MJ (min = 11.5 MJ, max = 46.3 MJ; Figure 3B).

**Figure 4:**
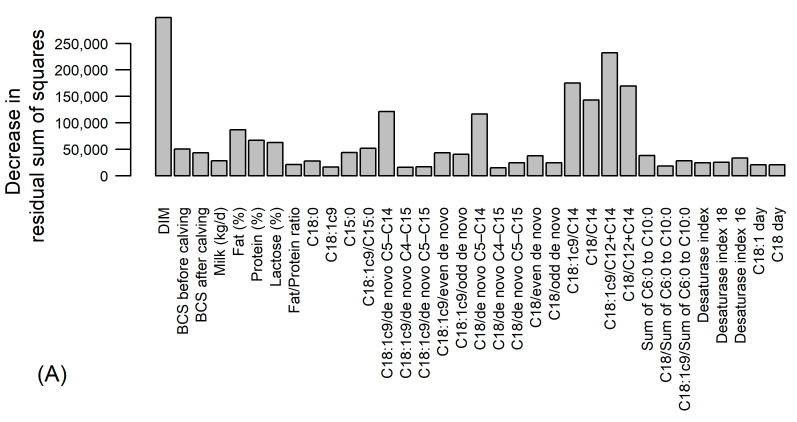

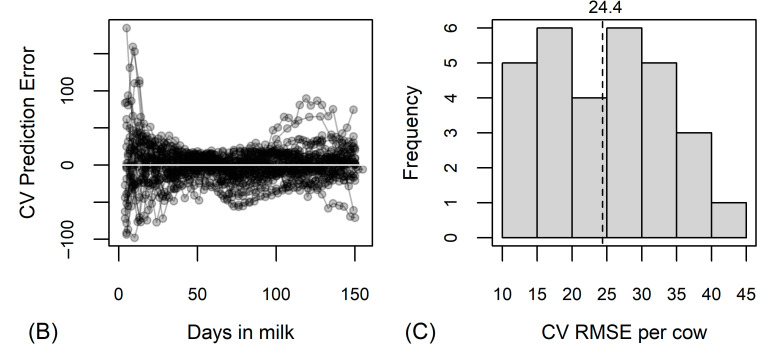
Results of random forest analysis predicting energy balance in MJ of effective energy based on 26 fatty acids and ratios, days in milk (DIM), body condition score (BCS) at calving and milk traits. (**A**) Variables’ importance according to the mean decrease in residual sum of squares from splitting on the variable; (**B**) cross-validated (CV) prediction error by cows (one line corresponds to observations of one cow) and days in milk; (**C**) distribution of cross-validated root mean square errors (CV RMSE) per cow, vertical dotted line with numerical value denotes the average CV RMSE.

The additional random forest analysis, including DIM, BCS at calving and milk traits, supported the results of the variance partitioning analysis—the average cross-validated RMSE per cow improved by only 3.1 MJ (Figure 4), verifying the results of the variance partitioning analysis. The inclusion of the somatic cell score as an independent variable did not change the prediction accuracy of the random forest.

**Figure A4:**
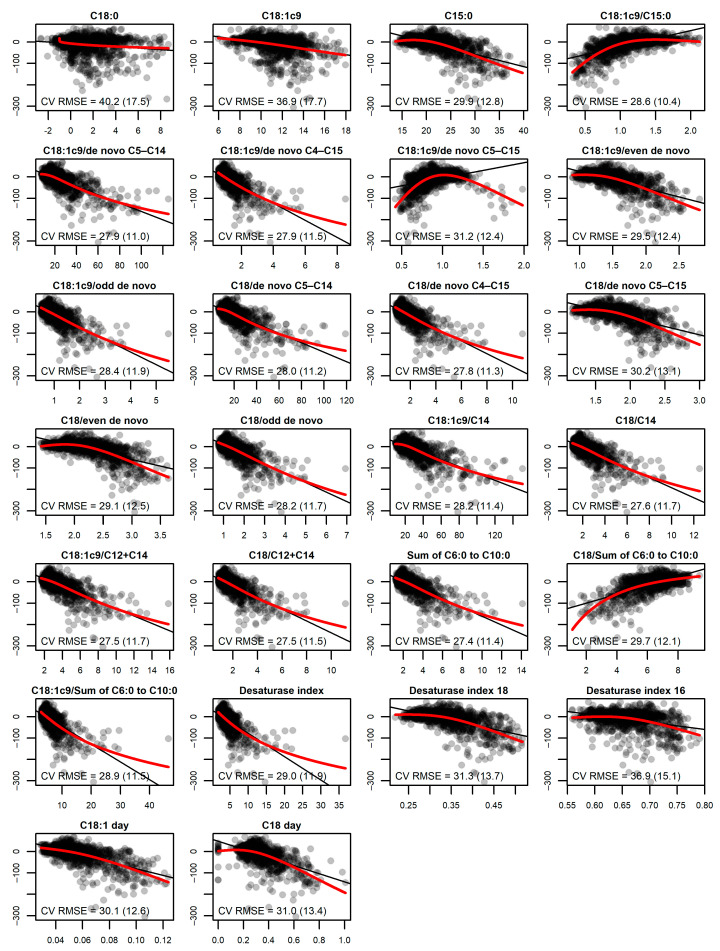
Relationship between energy balance (on vertical axis) and milk fatty acid groups and ratios (on horizontal axis) modelled with a natural cubic spline. The black narrow line denotes the overall linear relationship, and the red line denotes the fitted curve. The average cross-validated root mean square error (CV RMSE) with its standard deviation is presented numerically.

Using the general linear mixed model with the 26 FA studied, the mean cross-validated EB RMSE per cow varied within the interval 27.4–40.2 MJ (Appendix A Figure A4).

## 4. Discussion

This experiment was set up with dairy cows that had a range of BCS at calving with the aim to include animals with variations in the depth and time of the nadir of postpartum NEB. The trends in the estimated EB curves were as expected [23,24,25].

The variance partitioning analysis showed that FA and their ratios alone explained 63.6% of the variance, and other data obtained during this study increased the proportion of EB variance by only 3.5%, indicating the possibility to use milk FA and their ratios as sole predictors for EB variance. Similar to our results, Mäntysaari et al. [26] found that milk FA alone were better predictors of cow EB than milk and body traits. Also, adding the cow as a dummy variable did not significantly improve the proportion of EB variance covered, which indicates that the already treated factors, especially FA and their ratios, accounted for most of the cows’ individuality.

FA are grouped differently across studies, and discussion about the exact origin of some of them is ongoing. For example, C4:0 can be derived from condensation of acetyl units or directly from β-hydroxybutyrate obtained from blood [27]. Similarly, C15:0 and C17:0 can originate from rumen microbes and derive to milk fat from the blood or are synthesized de novo in the mammary gland from propionate as a primer; postruminal elongation of some BCFA have also been established [28]. In addition, the ratio of long-chain FA in milk, originating partly from body fat (C18:0 and C18:1cis9), can be affected by genetic parameters [29] and lactation stage [30]. It has raised the question if using the sum of substrate and resulting FA in milk FA models adds accuracy to the predictions. Since FAs could be assigned to various groups by origin, different combinations of FA, their groups and ratios were targeted in the present study (Table 1).

Elevated levels of C18:1cis9 concentration in milk fat has been previously considered a possible marker for NEB [11,12], increased blood plasma NEFA [13] and subclinical ketosis [31], but neither concentration nor daily production of the aforementioned single FA were found to be as good contributors in the present study. The decrease in the ratio C18:1c9/C14 during lactation was noted by Craninx et al. [32]. C18:1cis9/C15:0 was suggested as a possible hyperketonemia marker by Jorjong et al. [14] and for prediction of NEFA levels by Dórea et al. [33]. Khiaosa-Ard et al. [34] associated the ratios of 15:0/18:1cis9 and 14:0/18:1cis9 and the sum of de novo FA to 18:1cis9 with serum NEFA and BHB concentrations. In our previous study, we showed that the ratio of C18:1c9/C12:0+C14:0 was associated with increased blood plasma NEFA and BHB concentrations [35]. In the present study, C18:1c9/C12:0+C14:0 and C18:1c9+C18:0/C12:0+C14:0 showed the greatest reduction in the prediction errors in the regression trees, suggesting the sum of C12:0 and C14:0, though C14:0 contributes more to the sum, being more suitable to be used in models. Heck et al. [36] suggested that C12:0 is not completely synthesized de novo but can also originate from feed and be obtained from milk fat from blood plasma. No palm-based or coconut oil-based feeds rich in C12:0 were used in the dairy cow diets in the present study, but it should be taken into consideration in the future when predictions are made based on milk FA profiles.

The greater prediction errors in the first weeks of lactation and in the last third of the study may have been caused by the incorrect EB values calculated based on BW and BCS—the over-estimated EB values in these periods corresponded to the cows with the lowest calculated EB values. The assumption that residual gut fill is constant, used in the model [21], could have been one issue increasing the noise in the EB data [37] and led to bigger prediction errors in the last third of the study period.

The general linear mixed model analysis revealed that all studied 26 FA and their ratios predicted the EB with quite similar accuracy (Appendix A Figure A4). The similar prediction accuracy is logical because all studied FA and their groups were selected according to their potential relationship with EB, and they are all strongly related. However, the smallest CV RMSE in predicting the EB was achieved using C18/C14, C18:1c9/C12+C14, C18/C12+C14, C18:1c9/C14, C18/de novo C5–C14, C18:1c9/de novo C5–C14, C18:1c9/C15 and C18/even de novo. Most of the milk FA ratios best predicting EB were the same, which indicated the biggest decrease in prediction errors in the regression trees in the random forest analysis.

## 5. Conclusions

The results suggest that, of the studied predictors, milk FA composition may be a potential sole predictor of energy status in dairy cows. Random forest analysis highlighted the possibility to develop EB prediction models for dairy cows based on the ratios of selected blood-derived and de novo synthesized fatty acids. The best predictive potential has the ratios C18/C14, C18:1c9/C12+C14, C18/C12+C14, C18:1c9/C14, C18/de novo C5–C14, C18:1c9/de novo C5–C14, C18:1c9/C15 and C18/even de novo.

## Figures and Tables

**Table 1:** Fatty acids, fatty acid groups and ratios used in this study.

Fatty Acid Group	Fatty Acids
C18:0	C18:0
C18:1c9	C18:1cis9
C15:0	C15:0
C18:1c9/C15:0	C18:1cis9/C15:0
C18:1c9/de novo C5–C14	C18:1cis9/∑ (C5:0, C6:0, C7:0, C8:0, C9:0, C10:0, C10:1cis9, C11:0, C12:0, C12:1cis9, C13:0, C14:0, C14:1cis9)
C18:1c9/de novo C4–C15	C18:1cis9/∑ (C4:0, C5:0, C6:0, C7:0, C8:0, C9:0, C10:0, C10:1cis9, C11:0, C12:0, C12:1cis9, C13:0, C14:0, C14:1cis9, C15:0, C15:1cis9)
C18:1c9/de novo C5–C15	C18:1cis9/∑ (C5:0, C6:0, C7:0, C8:0, C9:0, C10:0, C10:1cis9, C11:0, C12:0, C12:1cis9, C13:0, C14:0, C14:1cis9, C15:0, C15:1cis9)
C18:1c9/even de novo	C18:1cis9/∑ (C4:0, C6:0, C8:0, C10:0, C10:1cis9, C11:0 C12:0, C12:1cis9, C14:0, C14:1cis9)
C18:1c9/odd de novo	C18:1cis9/∑ (C5:0, C7:0, C9:0, C13:0, C15:0, C15:1cis9)
C18/de novo C5–C14	∑ (C18:0, C18:1cis9)/∑ (C5:0, C6:0, C7:0, C8:0, C9:0, C10:0, C10:1cis9, C11:0, C12:0, C12:1cis9, C13:0, C14:0, C14:1cis9)
C18/de novo C4–C15	∑ (C18:0, C18:1cis9)/∑ (C4:0, C5:0, C6:0, C7:0, C8:0, C9:0, C10:0, C10:1cis9, C11:0, C12:0, C12:1cis9, C13:0, C14:0, C14:1cis9, C15:0, C15:1cis9)
C18/de novo C5–C15	∑ (C18:0, C18:1cis9)/∑ (C5:0, C6:0, C7:0, C8:0, C9:0, C10:0, C10:1cis9, C11:0, C12:0, C12:1cis9, C13:0, C14:0, C14:1cis9, C15:0, C15:1cis9)
C18/even de novo	∑ (C18:0, C18:1cis9)/∑ (C4:0, C6:0, C8:0, C10:0, C10:1cis9, C11:0, C12:0, C12:1cis9, C14:0, C14:1cis9)
C18/odd de novo	∑ (C18:0, C18:1cis9)/∑ (C5:0, C7:0, C9:0, C13:0, C15:0, C15:1cis9)
C18:1c9/C14	C18:1cis9/∑ (C14:0, C14:1cis9)
C18/C14	∑ (C18:0, C18:1cis9)/∑ (C14:0, C14:1cis9)
C18:1c9/C12+C14	C18:1cis9/∑ (C12:0, C12:1cis9, C14:0, C14:1cis9)
C18/C12+C14	∑ (C18:0, C18:1cis9)/∑ (C12:0, C12:1cis9, C14:0, C14:1cis9)
Sum of C6:0 to C10:0	∑ (C6:0, C8:0, C10:0)
C18/Sum of C6:0 to C10:0	∑ (C18:0, C18:1cis9)/∑ (C6:0, C8:0, C10:0)
C18:1c9/Sum of C6:0 to C10:0	C18:1cis9/∑ (C6:0, C8:0, C10:0)
Desaturase index	∑ (C12:1cis9, C14:1cis9, C15:1cis9; C16:1cis9, C17:1cis9, C18:1cis9)/∑ (C12:0, C12:1cis9, C14:0, C14:1cis9, C15:0, C15:1cis9; C16:0, C16:1cis9, C17:0, C17:1cis9, C18:0, C18:1cis9)
Desaturase index 18	C18:1cis9/∑ (C18:0, C18:1cis9)
Desaturase index 16	C16:1cis9/∑ (C16:0, C16:1cis9)
C18:1 day	Daily production of C18:1cis9
C18 day	Daily production of ∑ (C18:0, C18:1cis9)

## Data Availability

The data presented in this study are available on request from the corresponding author.
